# Fibrodysplasia Ossificans Progressiva Mimics Generalized Dystonia Disorder: A Case Report

**DOI:** 10.7759/cureus.50769

**Published:** 2023-12-19

**Authors:** Seraj Makkawi, Osama Khojah, Reema Abualnaja, Abdulaziz Qashqari, Nawaf A Alahmadi, Abdullatif G Bshnaq, Abdulrahman Alharthi, Hashem H Al-Hashemi, Aiman M Shawli

**Affiliations:** 1 College of Medicine, King Saud Bin Abdulaziz University for Health Sciences, Jeddah, SAU; 2 Research and Development, King Abdullah International Medical Research Center, Jeddah, SAU; 3 Neurosciences, Ministry of the National Guard-Health Affairs, Jeddah, SAU; 4 College of Medicine, King Saud bin Abdulaziz University for Health Sciences, Jeddah, SAU; 5 Department of Neurosciences, Ministry of the National Guard-Health Affairs, Jeddah, SAU; 6 Medicine, Ministry of the National Guard-Health Affairs, Jeddah, SAU; 7 Genetics and Precision Medicine, Ministry of the National Guard-Health Affairs, Jeddah, SAU

**Keywords:** stone man syndrome, munchmeyer disease, generalized dystonia disorder, myositis ossificans progressiva, fibrodysplasia ossificans progressiva

## Abstract

Fibrodysplasia ossificans progressiva (FOP) is an autosomal dominant disorder characterized by congenital deformities of the big toes and the progressive formation of extra-skeletal bone within soft tissues. The underlying genetic cause of FOP is mostly due to gain-of-function mutations in the AVCR1/ALK2 genes. These mutations cause aberrant bone morphogenetic protein (BMP) signaling pathways and eventually result in cumulative musculoskeletal impairment. FOP has a prevalence of approximately one in every 2 million people worldwide, with nearly 90% of patients being misdiagnosed, possibly leading to an underestimation of its true prevalence. To the best of our knowledge, there are only three reported cases in Saudi Arabia.

We report a case of a 21-year-old female patient, a product of a consanguineous marriage, referred to the neurology clinic for new-onset dysphagia and dysarthria in association with progressive painful muscle stiffness, which started at the age of four years. The diagnosis of generalized dystonia disorder was suspected, but eventually the whole exome sequencing showed a pathogenic missense mutation in the ACVR1 gene, confirming the diagnosis of FOP.

FOP is a rare, debilitating disorder that can be difficult to diagnose and manage. Current research efforts are focused on early diagnosis and a high index of suspicion to help prevent unnecessary investigations and procedures, slow the progression of the disease, and promote patients’ quality of life and long-term outcomes.

## Introduction

Fibrodysplasia ossificans progressiva (FOP) (Mendelian Inheritance in Man [MIM] #135100), also known as myositis ossificans progressiva or stone man syndrome, is a rare and debilitating hereditary connective tissue disorder characterized by progressive heterotopic ossification, a process that leads to atypical formation of bone in extraskeletal tissue, such as muscle and soft tissues [1]. Individuals with FOP appear normal at birth except for congenital malformations of the great toes, such as hallux valgus or monophalangism [2]. During their first decade of life, people with FOP develop painful inflammatory soft tissue swelling (flare-ups). These flare-ups can occur spontaneously or be triggered by minor trauma, injury, infections or immunization [3]. Although some flare-ups resolve on their own, most lead to the formation of heterotopic bone through endochondral ossification. This process transforms skeletal muscles, tendons, ligaments, fascia, and aponeuroses into mature heterotopic bone, resulting in severe joint restrictions and eventually permanent immobility [4]. Episodic ossification progresses in a distinct manner, mimicking the patterns of normal embryonic skeletal formation [5]. Initial episodes affect the dorsal, axial, cranial, and proximal regions of the body first [6]. The majority of FOP cases are attributed to autosomal dominant gain-of-function missense mutations in the ACVR1/ALK2 genes, which are responsible for encoding a bone morphogenetic protein (BMP) receptor [7]. This mutation triggers the continuous activation of the BMP signaling pathway, culminating in abnormal bone formation and the gradual development of a “second skeleton” [8]. FOP is considered an ultra-rare disorder with an estimated prevalence of one in every two million people worldwide and no known geographic, ethnic, racial, or gender predilection [1,9]. To date, more than 600 cases have been reported in the literature in various countries around the world, with only three cases in Saudi Arabia [10-13]. Nevertheless, it is worth noting that about 90% of FOP cases are misdiagnosed, leading to a possible underestimation of its true prevalence [14]. We describe a case of a 21-year-old female patient who was referred for swallowing difficulties and difficulty articulating with progressive restriction of movement in multiple joints.

## Case presentation

**Figure 1:**
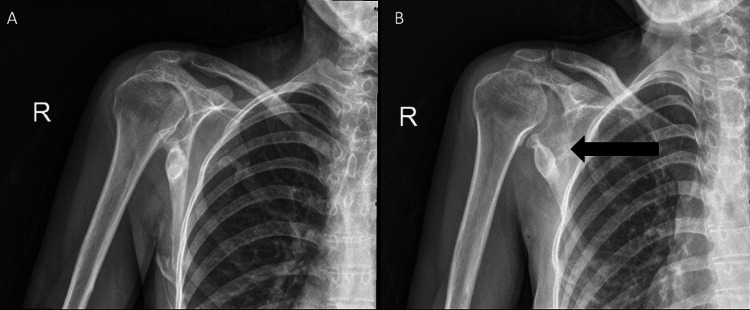
Shoulder X-ray (A, B) AP shoulder X-ray of the right performed before diagnosis showing mature heterotopic ossification (black arrows) and sclerosis of the scapula.

**Figure 2:**
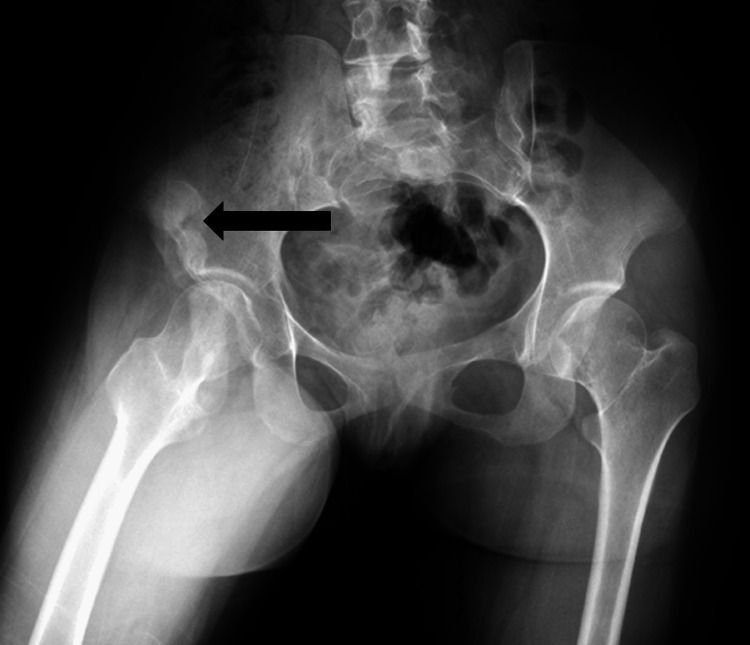
Pelvic X-ray AP pelvic X-ray showing sclerosis of the lateral border of the right iliac bone (black arrow) with pelvic tilt due to difficulty manipulating the joint

**Figure 3:**
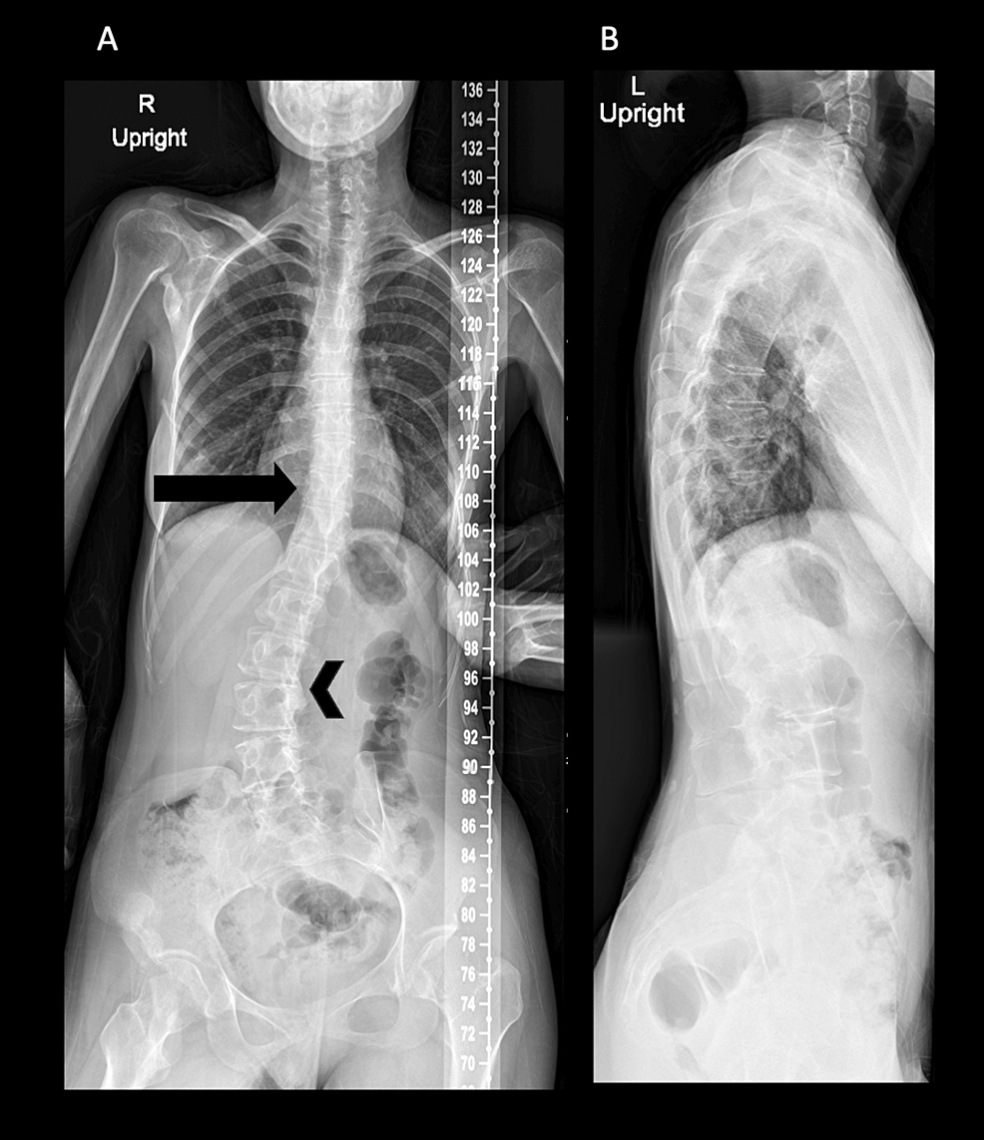
Scoliosis series X-ray (A) AP and (B) lateral scoliosis series X-ray showing gentle S-shaped scoliosis with concavity to the left at T11-T12 (black arrow) and to the right at the level of L2-L3 (arrowhead).

This is a 21-year-old female patient who was referred to the adult neurology clinic in King Abdulaziz Medical City, Jeddah, Saudi Arabia, for difficulties swallowing and articulating for six months associated with progressive restriction of movements of the neck, shoulder, elbow, hip, and knees and episodic tingling sensations over her lower limbs. Her past medical history is remarkable for painful muscle stiffness, which started at the age of four in the right shoulder and progressed over the years to the left upper limb. In the last two years, this has also involved the neck and lower limbs bilaterally, limiting her ability to ambulate. She is now completely reliant on her caretaker for daily activities. Her family history is remarkable for consanguineous parents without a similar disease in the family. The examination showed a patient with a scoliotic posture in a wheelchair who was oriented to time, place, and person. The patient had polyarticular stiffness in the majority of her body's joints, resulting in contractures and significant impairment. She had limitations in the following movements: neck flexion and extension, bilateral shoulder abduction and external rotation, bilateral elbow extension and flexion, right hip extension and flexion, and right knee extension with malformation of the halluces. Upper limb reflexes were preserved while the lower limb reflexes were absent, with absent upper motor neuron signs. Sensory examination was unremarkable in both upper and lower limbs. Previous imaging was reviewed and showed mature heterotopic ossification on shoulder X-ray (Figure 1), sclerosis of the right iliac bone (Figure 2), and S-shaped scoliosis on scoliosis series X-ray (Figure 3).

Initially, a generalized dystonia disorder was suspected based on the clinical presentation. Workups for genetic, metabolic, and structural causes were performed. Investigations showed normal serum copper (16.04 umol/L [normal range 11.78 to 22.77 umol/L]), ceruloplasmin (0.32 g/L [normal range 0.22 to 0.58 g/L]), creatine kinase (52 IU/L [normal range 27 to 132 IU/L]), pyruvate (0.08 mmol/L [normal range 0.03 to 0.08 mmol/L]), thyroid stimulating hormone (1.31 mIU/L [normal range 0.6 to 5.8 mIU/L]), free T4 (12.84 pmol/L [normal range 9 to 19 pmol/L]), but mildly elevated lactate dehydrogenase level (239 U/L [normal range 100 to 217 U/L]). WES (performed through our institution at a reference laboratory) showed a heterozygous missense pathogenic variant of the activin A receptor, type I (ACVR1) gene (c.617G>A p.(Arg206His)). The ultimate diagnosis was autosomal dominant fibrodysplasia ossificans progressiva. The NCS of the median, ulnar, radial tibial, common peroneal, and sural nerves of the right upper and lower limbs was unremarkable. Due to the nature of the disease and possible complications, an EMG was not performed. The patient was counseled regarding her condition and mode of inheritance.

## Discussion

Fibrodysplasia ossificans progressiva (FOP) is an uncommon genetic disorder affecting connective tissues. To the best of our knowledge, it has only been reported in three individuals in Saudi Arabia [11-13]. Our case shows that there is a FOP that could mimic many other diseases; there might be a significant delay in the diagnosis, which may subject the patient to disease-related complications.

In 1692, Guy Patin, a French physician, described the first patient and illustrated that she was “as hard as wood” [15]. Since then, hundreds of cases have been reported [16]. It is clear that, despite its distinct clinical picture, FOP is frequently diagnosed with difficulty and delay, and the disease remains a mystery to its medical attendants [17]. The differential diagnosis is wide and includes tumors (sarcomas, nodular fasciitis, osteoma cutis), infections/inflammations (tuberculosis, post-traumatic myositis, Weber-Christian disease), autoimmune diseases (rheumatoid arthritis, dermatomyositis, ankylosing spondylitis, systemic onset juvenile idiopathic arthritis), genetic diseases (Klippel-Feil syndrome, progressive osseous heteroplasia, Albright hereditary osteodystrophy, pseudohypoparathyroidism), and movement disorders (dystonia) [18-23]. In a questionnaire-based study on 138 patients with FOP, Kitterman et al. found that only 13% were initially diagnosed with FOP [14]. Our patient was initially referred to us as a case of neurogenic scoliosis, and after our examination, a diagnosis of generalized dystonia was suspected. Similar to our case, Subasree et al. reported a case of FOP initially mimicking cervical dystonia [18]. They reported an eight-year-old male whose neck, shoulder, and abdominal muscles were stiff and hard to move [18]. Similarly, Dulski and Sławek described a 34-year-old patient who was diagnosed with neck dystonia and treated with intravenous immunoglobulin and methylprednisolone with no improvement but benefited significantly from botulinum toxin A [24]. Patients presenting with contractions in multiple joints might mimic the posturing seen in patients with dystonia. Dystonia is a movement disorder that causes involuntary muscle contractions, which can lead to abnormal postures of any part or the entire body; this may be associated with pain and non-motor components [25]. Based on the part of the body involved, it is categorized into focal, segmental, multifocal, hemidystonia, and generalized dystonia. Patients’ symptoms may begin as focal dystonia, which progresses to generalized dystonia. In our patient, her symptoms started with progressive, painful muscle stiffness that started in the shoulder and eventually involved the extremities and neck. Delays in diagnosis not only cause management to be delayed, but they can also expose the patient to unnecessary interventions. Furthermore, an earlier diagnosis can make the patient aware of the precautions that must be taken. The debilitating effect of the disease should not be underestimated.

Management focuses on avoiding soft-tissue injuries, which can cause flare-ups. This includes biopsies, intramuscular injections, and surgery to prevent iatrogenic damage [2,26]. Following these principles, we did not find it necessary to perform an EMG on the patient after learning the diagnosis. EMG in FOP patients shows evidence of myopathy, while muscle biopsy typically reveals pathological changes indicative of a connective tissue disorder that primarily impacts the epimysium, tendons, and fasciae [27,28]. Bisphosphonates are medications that adsorb hydroxyapatite crystals, reducing the formation of abnormal bone during the active phase of the disease. In cases of severe movement restriction and gastric intolerance, intravenous bisphosphonate may be indicated and have a positive outcome [29]. It is important to note, however, that the disease's progression is unaffected. The patient can be educated about the role of mild physiotherapy at an early age, which may delay the progression of the disease [30].

The genetic basis of FOP is primarily due to spontaneous de novo mutations of the gene encoding activin receptor 1A/activin-like kinase 2 (ACVR1/ALK2), a type 1 receptor of the BMP signaling pathway. Most reported cases have been found to have a heterozygous missense mutation at the glycine-serine activation domain of ACVR1/ALK2 (c.617G>A). This mutation causes an amino acid change from arginine to histidine at the 206th position of the ACVR1 gene (R206H), leading to the dysregulated BMP signaling pathway observed in FOP. Although the majority of FOP cases exhibit the R206H mutation in ACVR1, there are phenotypic and genotypic variants of FOP patients worldwide. These variants involve mutations in other positions of the ACVR1 gene and are classified as atypical FOP. Additionally, all types of FOP involve highly conserved amino acids, which indicates their functional significance for the normal regulation of BMP signaling through the ACVR1 receptor.

While most cases of FOP are due to spontaneous mutations, there is evidence of autosomal dominant inheritance with complete penetrance in some individuals. In these cases, FOP can be inherited from either parent, or there is a 50% chance that the child of an affected person will have the condition. Inherited FOP may have variable phenotypic expression. Genetic counseling and the examination of both parents are crucial for understanding the risk of FOP in affected families. In our patient, a heterozygous pathogenic variant of the ACVR1 gene (c.617G>A; R206H) (NM_001105.4) was identified as the cause of FOP. Following this, the pathophysiology of FOP has been further elucidated by the efforts of research groups all over the world [31].

The involvement of the central nervous system (CNS) in otherwise classical muscle or connective tissue diseases is becoming more widely recognized. It has been proposed that FOP is a multisystemic disease that affects the CNS as well as the musculoskeletal system. This is based on imaging abnormalities such as demyelination and dentate nucleus hyperintensity observed in some patients [32]. Clinical examination and radiological imaging are used to identify any characteristic skeletal malformations associated with myositis ossificans progressive [33,34]. Magnetic resonance imaging (MRI), for example, is critical in making a diagnosis, particularly in the early stages of the disease [35]. Furthermore, Tc-99m bone scintigraphy can detect early ectopic ossification and provide valuable information about the disease's progression and extent [36]. The diagnosis is ultimately made through genetic testing to analyze the ACVR1/ALK2 genes [9]. 

## Conclusions

FOP is a rare and difficult disorder that can impair a patient's mobility and quality of life significantly. Prompt recognition, accurate diagnosis, and multidisciplinary care are required for effective management of this condition. Clinicians should be on the lookout for patients who have unexplained progressive musculoskeletal symptoms because early intervention can make a significant difference in the patient's long-term prognosis. Physicians dealing with dystonia should be aware of the possible differential diagnosis and the tests needed to reach the final diagnosis.
